# The effects of sintering temperature and duration on the flexural strength and grain size of zirconia

**DOI:** 10.3109/23337931.2015.1068126

**Published:** 2015-08-03

**Authors:** Nuri Murat Ersoy, Hasan Murat Aydoğdu, Beyza Ünalan Değirmenci, Neslihan Çökük, Müjde Sevimay

**Affiliations:** ^a^ Faculty of Dentistry, Department of Prosthodontics, Yüzüncü Yıl UniversityVanTurkey; ^b^ Faculty of Dentistry, Department of Prosthodontics, Selcuk UniversityKonyaTurkey

**Keywords:** Flexural strength, grain size, sintering time

## Abstract

*Objective*: This study investigated the effect of different sintering temperatures and times on the flexural strength and grain size of zirconia.

*Material and methods*: Zirconia specimens (In-Coris ZI, In-Coris TZI, 120 samples) were prepared in a partially sintered state. Subsequently, the specimens were randomly divided into three groups and sintered at different final sintering temperatures and for various durations: 1510 °C for 120 min, 1540 °C for 25 min and 1580 °C for 10 min. Three-point flexural strength (for 120 samples, 20 samples per group) was measured according to the ISO 6872: 2008 standards. The grain sizes were imaged by scanning electron microscopy (SEM) and the phase transitions were determined by X-ray diffraction (XRD). The data were analyzed using one-way ANOVA and Duncan tests (*p* < 0.05).

*Results*: The highest flexural strength was observed in ZI and TZI samples sintered at 1580 °C for 10 min. The differences between the ZI samples sintered at 1510 °C for 120 min and those sintered at 1540 °C for 25 min were statistically insignificant. Also, TZI samples sintered at 1510 °C for 120 min and those sintered at 1540 °C for 25 min also did not show any statistically significant differences. There were no visible differences in the grain sizes between the ZI and TZI specimens. The XRD patterns indicated similar crystalline structure for both materials subjected to the three different procedures.

*Conclusions*: The results of this study showed that experimented high sintering temperature and short sintering time combination increases the flexural strength of zirconia.

## Introduction

Zirconia is used for dental restoratives, such as crowns, bridges, implant fixtures and implant abutments [1] due to its suitable properties for dental prostheses.[2–4] The excellent mechanical properties of zirconia are attributed to the stress-induced transformation toughening mechanism, similar to that encountered in quenched steel.[5–7] Zirconia fixed partial dentures are used to replace posterior teeth because of the high flexural strength and fracture toughness of zirconia, which is used as the framework material.[8–10] Fractures of zirconia frameworks have rarely been reported.[11–15] In contrast, chipping of the veneering ceramic is a frequent complication.[11–15] From a clinical point of view, the stability of the system consisting of both the zirconia framework and the veneering ceramic is important.

To decrease the costs and simultaneously overcome the chipping issue, monolithic zirconia fixed dental prostheses without veneering ceramic are produced. Such restorations are esthetically unsuitable due to their high opacity. Sintering parameters have an effect on the crystalline content.[16–19] It has been shown that the holding time during sintering affects the grain growth in the material.[20] As the grain size increases, zirconia becomes less stable and more susceptible to spontaneous tetragonal-to-monoclinic phase transformations, which may result in a gradual strength decrease.[20]

The monoclinic phase is stable up to 1170 °C; above this temperature, it transforms into the tetragonal phase, which remains stable up to 2370 °C. The cubic phase of zirconia on the other hand, exists up to the melting point, 2680 °C.[21,22] The tetragonal form of metastable zirconia could be achieved at room temperature by alloying zirconia with other oxides (stabilizers), such as CaO,[23] MgO,[24] Y_2_O_3_,[25,26] and CeO_2_.[27] Y_2_O_3_ is the most widely used stabilizer for dental zirconia.[21] In response to tensile stresses at the crack-tips, the stabilized tetragonal zirconia transforms to the more stable monoclinic phase with a local volume increase of approximately 4–5%.[27] The differences in sintering parameters of zirconia can directly affect its microstructure and properties.[28] The extent of this effect have become of interest in the field of dental research especially after the introduction of short sintering cycles by manufacturers. Several authors have studied the effect of the changes in sintering time and temperature on the translucency, grain size and biaxial flexural strength of zirconia ceramics; however, the effect of these changes on the properties of zirconia remains in question.[29–32]

Computer-aided design and computer aided manufacturing (CAD/CAM) technologies enable the milling of zirconia into reconstructions with complex geometries. Two types of zirconia milling processes are currently available, i.e. soft-milling (partially sintered state) and hard-milling (full sintered). Soft-milled frameworks are subsequently sintered to full density. Different sintering parameters may show a strong influence on the properties of the zirconia frameworks.

Computer-aided design and computer aided manufacturing (CAD/CAM) chair side systems have reduced the operation times significantly and allowed for the production of most prosthetic restorations in one visit, although zirconia needs a sintering procedure, which takes several hours. Rapid sintering procedures, which can be carried out in minutes render the production of zirconia-based restorations possible in one visit and enhance its clinical use. Hence, translucent zirconia used for monolithic restorations was also included in this study. Use of translucent zirconia has the potential to eliminate delamination of the veneering ceramic, which has been known to be a common clinical problem and also reduce the amount of tooth preparation required.[33]

The aim of this study was to investigate the effect of different sintering temperatures and durations on the flexural strength, grain size and phase transformation of zirconia. The tested null hypothesis was that the decrease in final sintering time would decrease the flexural strength.

## Materials and methods

Sixty zirconium oxide and 60 highly translucent zirconium oxide bar specimens (In-Coris ZI and In-Coris TZI, Sirona Dental Systems GmbH, Bensheim, Germany) were cut using a low speed diamond saw (Isomet 1000, Buehler, IL). Then, the ZI and TZI specimens were randomly divided into three subgroups (with each group containing *n* = 20 samples) according to the sintering time and temperature. The employed parameters belong to fixed program numbers 1 – superspeed for groups C and F; 2 – speed for groups B and E; 5 – classic for groups A and D of the sintering furnace (inFire HTC Speed, Sirona, Bensheim Germany). The test groups are listed below.

Group A (ZI): Slow sintering program (regular); sintered at 1510 °C with a dwell time of 120 min. Total time is approximately 8 h.

Group B (ZI): Faster sintering program (speed); sintered at 1540 °C with a dwell time of 25 min. Total time is approximately 2 h.

Group C (ZI): Rapid sintering program (super speed); needs preheating to 1580 °C, starts at 1580 °C with a dwell time of 10 min. At the end of dwell time, furnace opens and material is immediately removed. Total time is 10 min.

Group D (TZI): Slow sintering program (regular); sintered at 1510 °C with a dwell time of 120 min. Total time is approximately 8 h.

Group E (TZI): Faster sintering program (speed); sintered at 1540 °C with a dwell time of 25 min. Total time is approximately 2 h.

Group F (TZI): Rapid sintering program (super speed); needs pre-heating to 1580 °C, starts at 1580 °C with a dwell time of 10 min. At the end of dwell time, furnace opens and material is immediately removed. Total time is 10 min (Table 1).

**Table 1.** Classification of the zirconia bar specimens to the groups according to sintering time.

Group	Material	Sintering temperature	Dwell time (min)	Total time
A	In-Coris ZI	1510 °C	120	8 h
B	In-Coris ZI	1540 °C	25	2 h
C	In-Coris ZI	1580 °C	10	10 min^a^
D	In-Coris TZI	1510 °C	120	8 h
E	In-Coris TZI	1540 °C	25	2 h
F	In-Coris TZI	1580 °C	10	10 min*

^a^Applied in pre-heated furnace while the others start from room temperature.

### Three-point flexural strength tests

Three-point flexural strength (total number of specimens, *N* = 120; number of samples per group *n* = 20) was measured according to the ISO 6872:2008 standards.[34] After sintering, the final dimensions of all the specimens were 1.2 mm × 4 mm × 25 mm. Before the flexural strength test, the dimensions of the specimens were measured with a digital micrometer (Absolute Digimatic Caliper, Mitutoyo, Tokyo, Japan) to an accuracy of 0.01 mm. The specimens were then placed in the appropriate sample holder and loaded in a Universal testing machine (Shimadzu Model AGS-X, Kyoto, Japan) at a crosshead speed of 1 mm/min until failure. The specimens were tested dry at room temperature. The flexural strength was calculated according to the following formula.

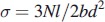



σ is the flexural strength, *N* is the fracture load (N), *l* is the distance between the supports (mm), *b* is the width of the specimen (mm) and *d* is the thickness of the specimen (mm).

### Zirconia grain size

After sintering, the surfaces of all the specimens (*N* = 6, *n* = 1 per group) were polished up to a thickness of 1 µm with a diamond suspension (Struers, Bellerup, Denmark) and ultrasonically cleaned in isopropanol. Then, the specimens were dried at 50 °C for 24 h (Nüve Incubator EN 120, Ankara, Turkey) and coated with 9 nm gold-palladium particles (Cressington sputtercoater 108 auto, Cressington MTM-20, Elektronen-Optik-Service, Dortmund, Germany) and the surface topography was evaluated using a scanning electron microscope (Evo LS10, Carl Zeiss, Oberkochen, Germany) at magnifications of 5000× and 15,000×.

### Crystal structure analysis

Three specimens were selected randomly from each subgroup for X-ray diffraction (XRD) surface analysis to detect the amount of tetragonal and monoclinic phases present. The specimens were placed in the holder of a diffractometer (Bruker D8 advance-Lynxeye detector) and irradiated with Cu Kα. The spectrum was recorded within the range of 0–80° at a scan time per step of 1 min. The voltage and current were set to 40 kV and 40 mA, respectively.

### Statistical analysis

The data collected was checked for normal distribution and analyzed using one-way analysis of variance (ANOVA), followed by Duncan tests (SAS; Statistical Analysis System, SAS Institute Inc., Cary, NC) at a significance level of *p* < 0.05 to determine the effect of sintering time and temperature on each of the variables tested.

## Results

The mean flexural strength values (MPa) and standard deviations for each group are presented in Table 2.

**Table 2.** Mean and standard deviations (SD) values of all tested groups.

Group	Mean (MPa)	sd
Group A	700.3^a^	125.3
Group B	662.1^a^	77.8
Group C	871.8^b^	108.8
Group D	579.7^a^	140.6
Group E	622.3^a^	82.7
Group F	904.2^b^	115.7

Letters rendered in superscripts represent a significant difference.

One-way ANOVA showed statistically significant differences between the groups (*p* < 0.05). The mean flexural strength of group C (ZI, 1580 °C sintering temperature and total 10 min sintering time) was significantly higher than group A and B. Also, the mean flexural strength of group F (TZI, 1580 °C sintering temperature and total 10 min sintering time) was significantly higher than group D and group E (Table 2).

**Figure 1.**
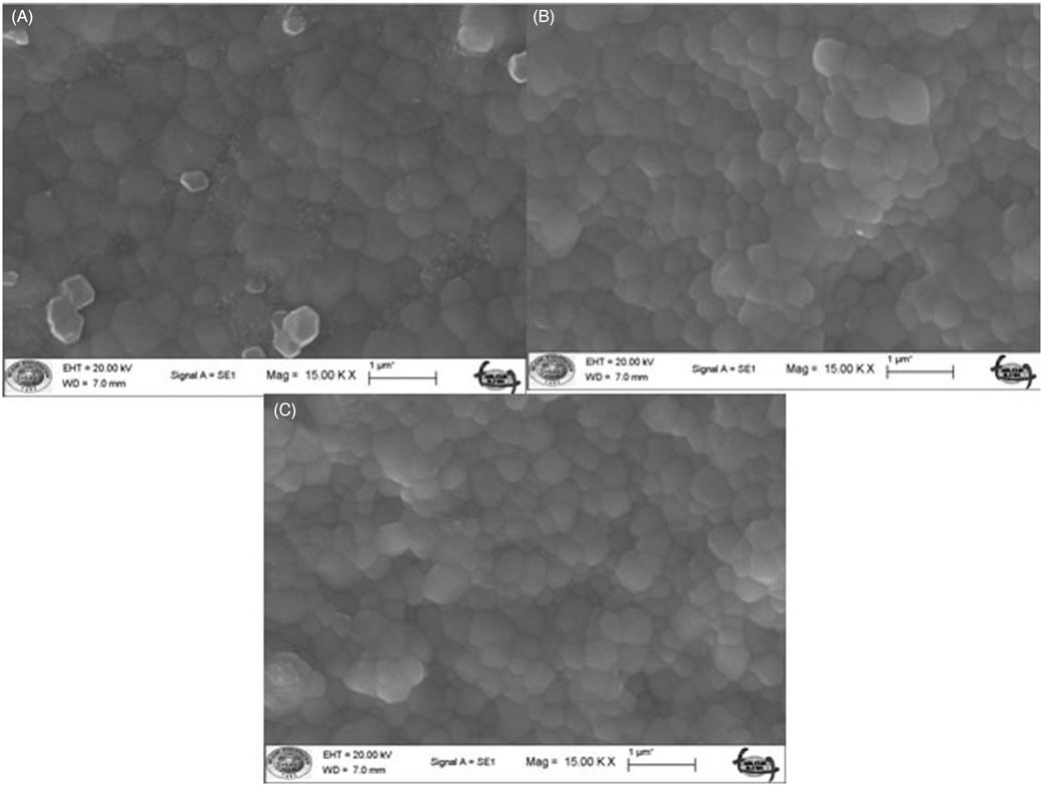
In-Coris ZI grain size after different sintering temperatures and times (15,000×): (A) 1510 °C with a dwell time of 120 min; (B) 1540 °C with a dwell time of 25 min; (C) 1580 °C with a dwell time of 10 min.

**Figure 2.**
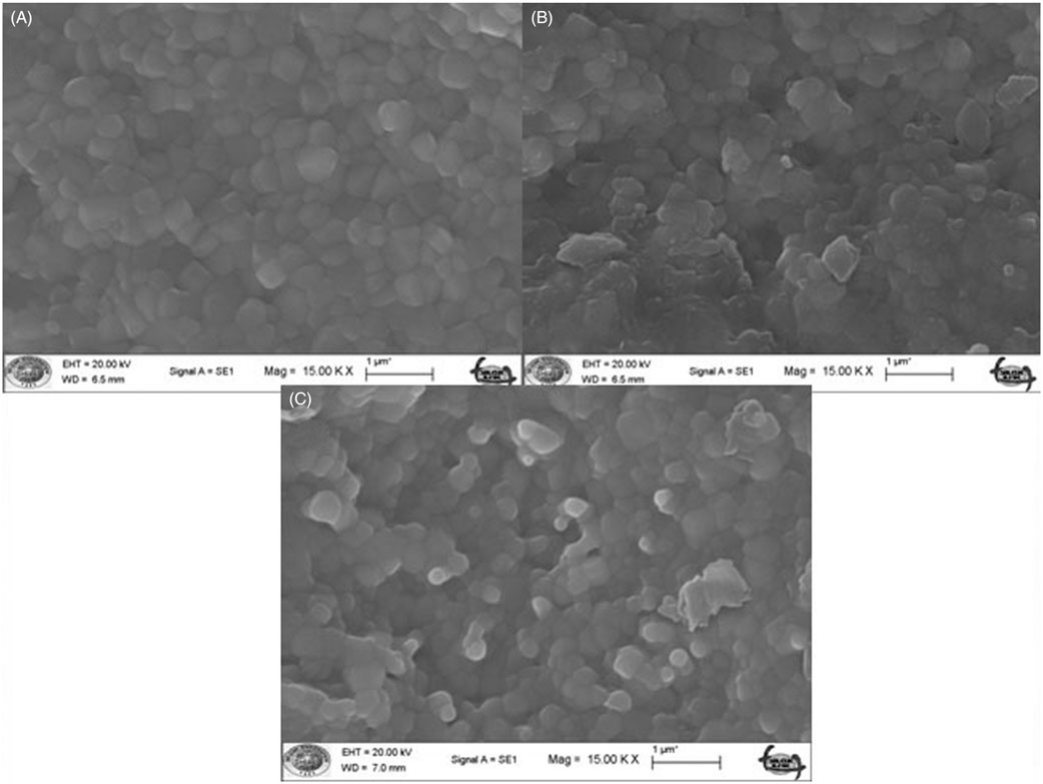
In-Coris TZI grain size after different sintering temperatures and times (15,000×): (A) 1510 °C with a dwell time of 120 min; (B) 1540 °C with a dwell time of 25 min; (C) 1580 °C with a dwell time of 10 min.

Scanning electron microscopy (SEM) images were used to observe zirconia grain sizes. (Figures 1 and 2). The difference in grain size caused by different sintering procedures is generally small and it is difficult to see if there is a difference. In this study, no quantitative analysis was done, for this reason we could not say there were no visible difference in the grain size between the ZI and TZI groups.

**Figure 3.**
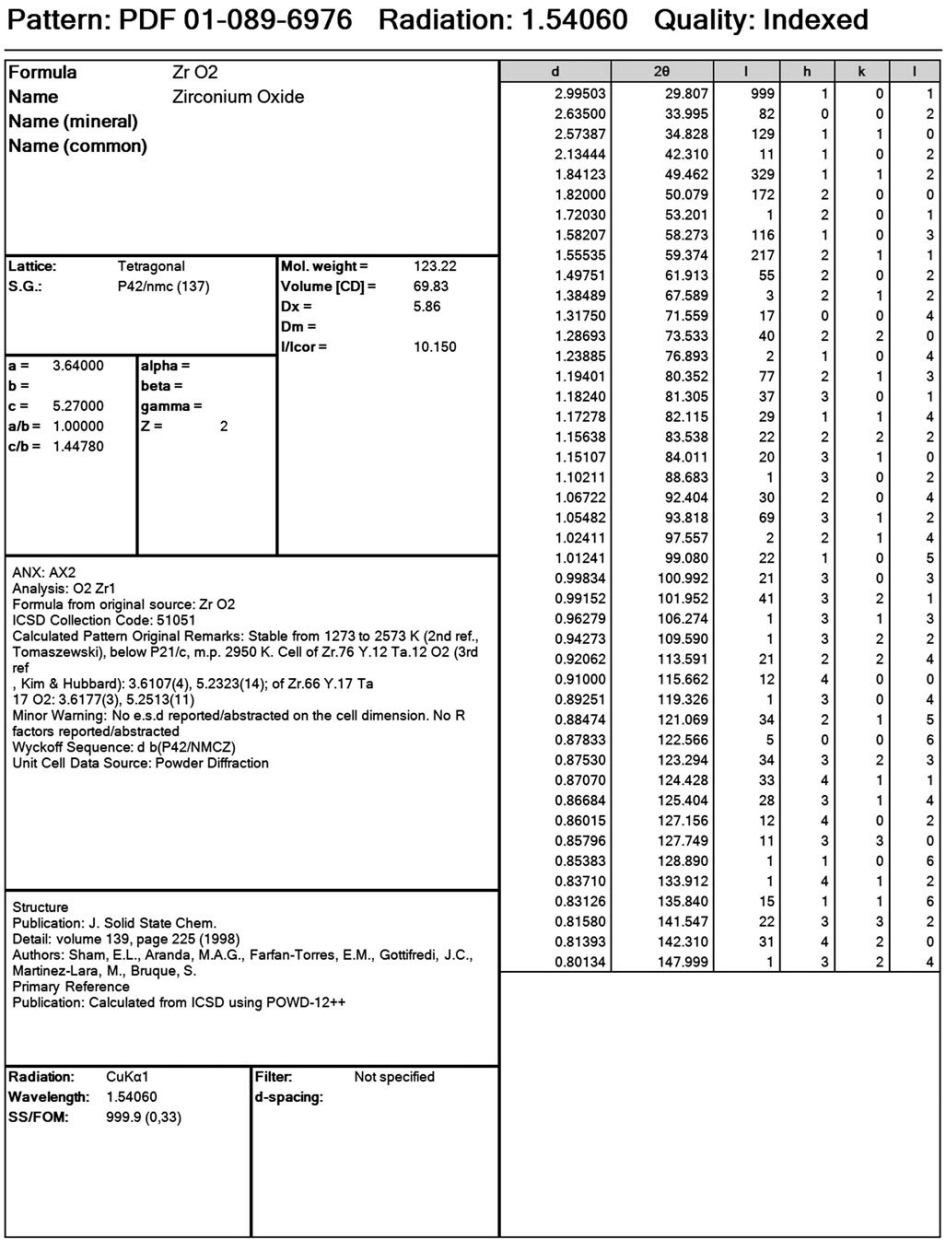
ICSD card for tetragonal phase for ZrO_2_.

**Figure 4.**
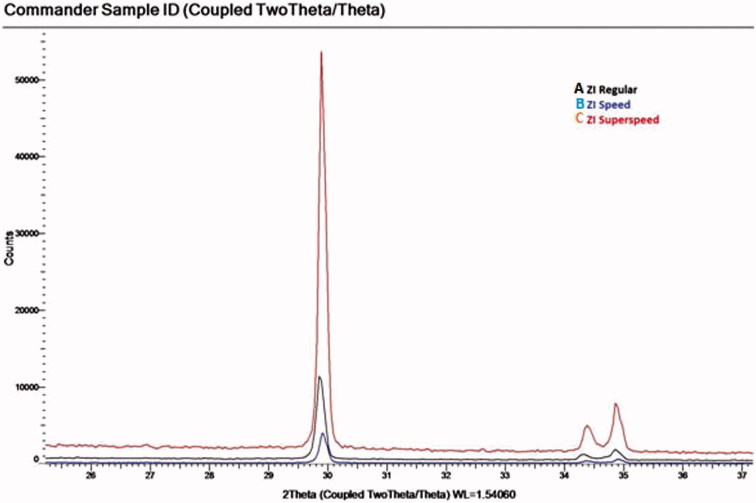
X-ray diffraction analysis of In-Coris ZI.

**Figure 5.**
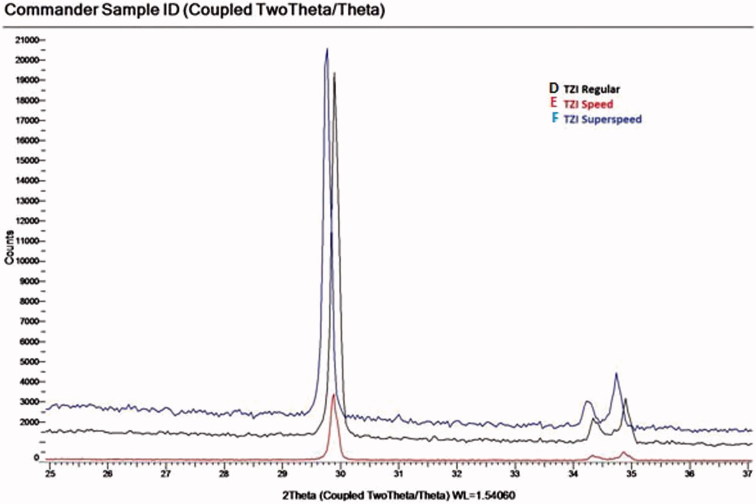
X-ray diffraction analysis of In-Coris TZI.

The microstructure analysis of the specimens using XRD revealed that the peak positions for the spectra of the samples matches the corresponding ICSD card for tetragonal phase for ZrO_2_ within the resolution of the data (PDF# 01-089-6976; (Figures 3–5).

## Discussion

The 1580 °C–10 min sintering program (C and F groups) led to the highest flexural strength values for both materials. For groups A, B, D and E, the sintering temperature and time combinations did not have a significant effect on the flexural strength of zirconia. Hence, the null hypothesis is rejected.

In our study, SEM images were used to observe the grain sizes visually. However, since the visual observation alone is not enough for comparing grain sizes, quantitative analyses should be done for the determination of differences. This study was undertaken to demonstrate the possible change in strength of sintered green bar-shaped zirconia specimens by varying the sintering conditions. The main motivation behind undertaking this study was that the properties of some green milled zirconia can reach higher strengths when the sintering temperature and time are altered. Also, questioning the total phase transformation from monoclinic to tetragonal during the 10 min sintering process is also of interest. Clinically, shorter sintering times would be also beneficial for the rapid manufacturing of zirconia-based prostheses.

It is to be noted that the mechanical analysis in this study uses static loading tests and dynamic (fatigue) tests would more closely resemble clinical masticatory forces. However, there is a correlation between static and fatigue properties, analogous to studies, which describe different damage modes or strength degradation, while comparing results of cyclic loading and monotonic loading tests.[35,36] Itinoche et al. [37] found marginal differences in the flexural strength of zirconia obtained by static and cyclic loading tests, although the differences were statistically insignificant.

Crystal structural analysis revealed that all subgroups contained typical tetragonal phase grains. All specimens were completely sintered to the tetragonal phase and did not transform back to the monoclinic phase. This was predicted in the light of the absence of any physical or thermal treatment on the specimens.

Stawarczyk et al. [30] reported that the grain size of zirconia increased with increase in sintering temperatures. They also reported that the sintering temperature showed a significant negative correlation with flexural strength and concluded that the sintering temperature for zirconia should be limited below 1550 °C. Our findings contradicts with their results, because we achieved higher flexural strength values with 1580 °C sintering. This difference can be explained by several reasons: the varying brands of zirconia used in both studies, our narrower temperature range and altering the sintering times with changing temperatures. It is to be noted that all our specimens were sintered as-milled and were unpolished; thus, comparing the flexural strength values obtained in this study to the values reported in other studies using polished specimens is questionable.

The flexural strength of the groups sintered at 1580 °C with a dwell time of 10 min (speed groups) were found to be significantly higher than that exhibited by the other groups (speed and regular groups) used in this study. Hjerppe et al. [38] investigated the interaction between the sintering time and static biaxial flexural strength of zirconia. According to their results, shorter sintering times did not affect the biaxial flexural strength, while having correlation to the surface composition of the samples. This can affect the durability of zirconia after water exposure, which can be clinically significant for monolithic zirconia restorations uncovered by porcelain intraorally. In our study, shorter sintering times affected flexural strength. We did not analyze the surface composition of samples; this analysis can be helpful for explaining such difference.

This study has limitations. First, only one brand of zirconia was used. The results may not be applicable for other brands with different grain sizes and different manufacturers may have special recommendations for sintering zirconia. Sintering with shortened dwell time was also influenced the density of the material but in the current study the effect of sintering on the density of the material was not studied. Further, we have used static *in vitro* tests; however, dynamic fatigue tests are more representative of clinical masticatory forces and further *in vitro* and *in vivo* tests are required. Kim et al. [29] recommended that the physical properties and marginal fitness of the coping are to be analyzed in relation to the sintering method, grain size and light transmittance. We have studied the physical properties related to the sintering method and the marginal fit and light transmittance of zirconia in correlation to the sintering method may require further investigations.

## Conclusions

Based on the results obtained in this study, the following conclusions can be drawn.

1. Zirconia samples tested showed the highest flexural strength when sintering was carried out at 1580 °C for 10 min.
2. All experimented sintering parameters have provided full sinterization for green zirconia.

## References

[1] Ban S Reliability and properties of core materials for all-ceramic dental restorations. Japan Dent Sci Rev. 2008;44:3–21

[2] Bruder SP, Jaiswal N, Haynesworth SE Growth kinetics, self-renewal, and the osteogenic potential of purified human mesenchymal stem cells during extensive subcultivation and following cryopreservation. J Cell Biochem. 1997;64:278–294 9027588 10.1002/(sici)1097-4644(199702)64:2<278::aid-jcb11>3.0.co;2-f

[3] Sato H, Yamada K, Pezzotti G, Nawa M, Ban S Mechanical properties of dental zirconia ceramics changed with sandblasting and heat treatment. Dent Mater. 2008;27:408–414 10.4012/dmj.27.408 18717169

[4] Yamashita D, Machigashira M, Miyamoto M, Takeuchi H, Noguchi K, Izumi Y, et al Effect of surface roughness on initial responses of osteoblast-like cells on two types of zirconia. Dent Mater. 2009;28:461–470 10.4012/dmj.28.461 19721284

[5] Okuda Y, Noda M, Kono H, Miyamoto M, Sato H, Ban S Radio-opacity of core materials for all ceramic restorations. Dent Mater. 2010;29:35–40 10.4012/dmj.2009-54 20379010

[6] Ban S, Suehiro Y, Nakanishi H Fracture toughness of dental zirconia before and after autoclaving. J Ceram Soc Jpn. 2010;118:406–409

[7] Noda M, Okuda Y, Tsuruki J, Minesaki Y, Takenouchi Y, Ban S Surface damages of zirconia by Nd:YAG dental laser irradiation. Dent Mater. 2010;29:536–541 10.4012/dmj.2009-127 20877130

[8] Hannink RHJ, Kelly PM, Muddle B Transformation toughening in zirconia-containing ceramics. J Am Ceram Soc. 2000;83:461–487

[9] Fischer J, Stawarczyk B Compatibility of machined Ce-TZP/Al_2_O_3_ nanocomposite and a veneering ceramic. Dent Mater. 2007;23:1500–1505 17376524 10.1016/j.dental.2007.01.005

[10] Aboushelib MN, Feilzer CJ, Feilzer AJ Evaluation of a high fracture toughness composite ceramic for dental applications. J Prosthodont. 2008;17:538–544 18761572 10.1111/j.1532-849X.2008.00346.x

[11] Vult von Steyern PV, Carlson P, Nilner K All-ceramic fixed partial dentures designed according to the DC-Zircon technique: a 2-year clinical study. J Oral Rehabil. 2005;32:180–187 15707428 10.1111/j.1365-2842.2004.01437.x

[12] Raigrodski AJ, Chiche GJ, Potiket N, Hochstedler JL, Mohamed SE, Billiot S, et al The efficacy of posterior -unit zirconium-oxide-based ceramic fixed partial dental prostheses: a prospective clinical pilot study. J Prosthet Dent. 2006;96:237–244 17052467 10.1016/j.prosdent.2006.08.010

[13] Sailer I, Fehér A, Filser F, Gauckler LJ, Lüthy H, Hämmerle Ch Five-year clinical results of zirconia frameworks for posterior fixed partial dentures. Int J Prosthodont. 2007;20:383–388 17695869

[14] Edelhoff D, Florian B, Florian W, Johnen C HIP zirconia fixed partial dentures – clinical results after 3 years of clinical service. Quintessence Int. 2008;39:459–471 19057742

[15] Schmitt J, Holst S, Wichmann M, Reich S, Gollner M, Hamel J Zirconia posterior-fixed partial dentures: a prospective clinical-year follow-up. Int J Prosthodont. 2009;22:597–603 19918596

[16] Heffernan MJ, Aguilino SA, Diaz-Arnold AM, Haselton DR, Stanford CM, Vargas MA Relative translucency of six all-ceramic systems. Part I: core materials. J Prosthet Dent. 2002;88:4–9 12239472

[17] Kelly JR, Nishimura I, Campbell S Ceramics in dentistry: historical roots and current perspectives. J Prosthet Dent. 1996;75:18–32 9005250 10.1016/s0022-3913(96)90413-8

[18] Chen YM, Smales RJ, Yip KH, Sung WJ Translucency and biaxial flexural strength of four ceramic core materials. Dent Mater. 2008;24:1506–1511 18440062 10.1016/j.dental.2008.03.010

[19] Tsukuma K, Kubota Y, Tsukidate T Thermal and mechanical properties of Y_2_O3-stabilized tetragonal zirconia polycrystals. In: Clausen N, Ruehle M, Heuer AH, editors. Science and technology of zirconia II. Columbus, OH: The American Ceramic Society;1984 p. 382–390

[20] Matsui K, Yoshida H, Ikuhara Y Isothermal sintering effect on phase separation and grain growth in yttria-stabilized tetragonal zirconia polycrystal. J Am Ceram Soc. 2009;92:467–475

[21] Kisi EH, Howard CJ Crystal structure of zirconia phases and their inter-relation. Key Eng Mater. 1998;153:1–36

[22] Lughi V, Sergo V Low temperature degradation aging of zirconia: a critical review of the relevant aspects in dentistry. Dent Mater. 2010;26:807–820 20537701 10.1016/j.dental.2010.04.006

[23] Fassina P, Zaghini N, Bukat A, Piconi C, Greco F, Piantelli S Yttria and calcia partially stabilized zirconia for biomedical applications. In: Ravagliogli A, Krajewski A, editors. Bioceramics and the human body. London and New York: Elsevier Applied Science; 1992 p. 223–229

[24] Garvie RC, Urbani C, Kennedy DR Biocompatibility of magnesia partially stabilized zirconia (mg-PSZ) ceramics. J Mater Sci. 1984;19:3224–3228

[25] Chevalier J, Deville S, Munch E, et al Critical effect of cubic phase on aging in 3 mol% yttria-stabilized zirconia ceramics for hip replacement prosthesis. Biomaterials. 2004;25:5539–5545 15142736 10.1016/j.biomaterials.2004.01.002

[26] Deville S, Gremillard L, Chevalier J, et al A critical comparison of methods for the determination of the aging sensitivity in biomedical grade yttria-stabilized zirconia. J Biomed Mater Res B Appl Biomater. 2005;72:239–245 15654702 10.1002/jbm.b.30123

[27] Studart AR, Filser F, Kocher P, Gauckler LJ Fatigue of zirconia under cycling loading in water and its implications for the design of dental bridges. Dent Mater. 2007;23:106–114 16473402 10.1016/j.dental.2005.12.008

[28] Chevalier J What future for zirconia as a biomaterial? Biomaterials. 2006;27:535–543 16143387 10.1016/j.biomaterials.2005.07.034

[29] Kim MJ, Ahn JS, Kim JH, Kim HY, Kim WC Effects of the sintering conditions of dental zirconia ceramics on the grain size and translucency. J Adv Prosthodont. 2013;5:161–166 23755342 10.4047/jap.2013.5.2.161 PMC3675289

[30] Stawarczyk B, Özcan M, Hallmann L, Ender A, Mehl A, Hammerlet Ch The effect of zirconia sintering temperature on flexural strength, grain size, and contrast ratio. Clin Oral Invest. 2013;17:269–274 10.1007/s00784-012-0692-6 22358379

[31] Hjerppe J, Narhi T, Froberg K, Vallittu PK, Lassila LV Effect of shading the zirconia framework on biaxial strength and surface microhardness. Acta Ododntol Scand. 2008;66:262–267 10.1080/00016350802247123 18645687

[32] Jiang L, Liao Y, Wan Q, Li W Effects of sintering temperature and particle size on the translucency of zirconium dioxide dental ceramic. J Mater Sci Mater Med. 2011;22:2429–2435 21922331 10.1007/s10856-011-4438-9

[33] Sailer I, Fehér A, Filser F, Lüthy H, Gauckler LJ, Schärer P, Franz Hämmerle Ch Prospective clinical study of zirconia posterior fixed partial dentures: 3-year follow-up. Quintessence Int. 2006;37:685–693 17017630

[34] ISO 6872: 2008 Dentistry – Ceramic materials

[35] Zhou J, Mah J, Shrotriya P, Mercer C, Soboyejo Wh Contact damage in an yttria stabilized zirconia: implications for biomedical applications. J Mater Sci Mater Med. 2007;18:71–78 17200816 10.1007/s10856-006-0664-y

[36] Jung YG, Peterson IM, Kim DK, Lawn BR Lifetime limiting strength degradation from contact fatigue in dental ceramics. J Dent Res. 2000;79:722–731 10728973 10.1177/00220345000790020501

[37] Itınoche KM, Özcan M, Bottino MA, Oyafuso D Effect of mechanical cycling on the flexural strength of densely sintered ceramics. Dent Mater. 2006;22:1029–1034 16631923 10.1016/j.dental.2005.11.025

[38] Hjerppe J, Vallittu PK, Fröberg K, Lassila LV Effect of sintering time on biaxial strength of zirconium dioxide. Dent Mater. 2009;25:166–171 18632146 10.1016/j.dental.2008.05.011

